# The burden of acute eye conditions on different healthcare providers in Wales: a retrospective population-based study

**DOI:** 10.23889/ijpds.v9i5.2561

**Published:** 2024-09-18

**Authors:** Rachel Huck, Sarah Cummins

**Affiliations:** 1 Office for National Statistics

## Objective

Ordinarily data linkage is approached on a project-by-project basis, tailoring the method to the research need. An increase in demand for data linkage has led to the development of a more efficient strategy, i.e. a generalised approach.

Through data linkage, a series of indexes representing people, businesses, geography and classifications are created using administrative data. Additional data are linked to the indexes via a general method to collect a common identifier.

Adopting a generalised approach requires an evidence base to ensure research aims can be met by the methods. This research will provide the evidence needed to underpin the strategy as well as guidance for implementing a generalised approach using an index.

## Approach

The existing data linkage portfolio provides an opportunity to compare the application of a bespoke to a generalised methodology. Comparisons will collate information on:

- Index coverage;
- Linkage results and quality including precision and recall estimates;
- Analysis of the bias observed within the linked data.

Of particular interest is the linkage of different population sub-groups, particularly those classified as vulnerable or disadvantaged, for example, data for prisoners, refugees, the homeless and third-sector organisations.

## Results

We will present the findings to date on recommendations for the use of a generalisable method for indexing, by exploring:

- Data linkage quality by bespoke methods versus a generalised approach
- The coverage strengths/limitations of the indexes
- Sub-groups representation in the linked data

## Conclusions/Implications

Findings will provide evidence for adopting a new data linkage strategy, ensuring the most appropriate data linkage methods and quality assurance have been applied. Therefore, producing accurate and unbiased analysis to form the evidence base for policy decisions.

